# AI-Guided Radiomics for Spectral-Domain OCT Characterization of Acquired Vitelliform Lesions: A Pilot Study

**DOI:** 10.3390/jcm15218269

**Published:** 2026-10-23

**Authors:** Marina Concilio, Gilda Cennamo, Renato Cuocolo, Clementina Miele, Michele Rinaldi, Ciro Costagliola

**Affiliations:** 1 Department of Neurosciences, Reproductive Sciences and Dentistry, University of Naples “Federico II”, 80138 Napoli, Italy; conciliomarina@gmail.com (M.C.); michrinaldi@libero.it (M.R.); ciro.costagliola@unina.it (C.C.); 2 Department of Medicine, Surgery and Dentistry, University of Salerno, 84081 Baronissi, Italy; rcuocolo@unisa.it (R.C.); cmiele@unisa.it (C.M.)

**Keywords:** vitelliform lesion, SD-OCT, radiomics, artificial intelligence

## Abstract

**Objective**: To evaluate the ability of artificial intelligence (AI)-driven radiomics applied to structural OCT to characterize hypo-reflective subretinal spaces in acquired vitelliform lesions (aVLs), to distinguish reabsorbed material from subretinal fluid, improving diagnostic precision. **Methods**: In this retrospective monocentric study, patients with aVLs were compared with a control group of acute central serous chorioretinopathy (CSCR) to provide reference OCT patterns for subretinal fluid. All participants underwent multimodal imaging. OCT scans were manually segmented. Radiomic features were extracted using a standardized pipeline, including image preprocessing, robustness assessment, variance and correlation filtering, and feature selection with a WEKA subset evaluator. A ridge-based Logistic Regression classifier was trained on 75% of the dataset and tested on the remaining 25%. Model performance was assessed using accuracy metrics, ROC AUC, and precision–recall curves. **Results**: A total of 520 radiomics features were extracted, of which nine were selected for training. The classifier achieved an accuracy of 86.5% in the training set (AUC 0.89; PRC 0.86) and 80% in the independent test set, with excellent AUC (0.92) and PRC (0.92). The negative predictive value reached 92%, indicating a high ability to correctly classify the aVL-associated hypo-reflective phenotype. The positive predictive value was 67%. **Conclusions**: AI-based radiomics demonstrated promising performance in discriminating, on structural OCT, between hypo-reflective subretinal spaces associated with the resorptive phase of aVLs and subretinal fluid. These preliminary findings support the feasibility of quantitative radiomic characterization of these two OCT phenotypes. Further studies are required to determine whether this approach may also be useful in identifying neovascular complications.

## 1. Introduction

The term “vitelliform lesions” (VLs) refers to the presence of a “dome-shaped” visible yellowish material at the fundus oculi and/or on fundus photography (FP), corresponding to hyperautofluorescent material on fundus autofluorescence (FAF) and the presence of subretinal hyperreflective material (SHRM) with or without associated subretinal fluid (SRF) on structural optical coherence tomography (OCT) examination [1]. The causes of VLs are varied: they may be hereditary or, more commonly, sporadic and occur in a wide range of age groups [2]. In young people, the etiology is Best disease, which is inherited in an autosomal dominant manner and is most often associated with a mutation in the BEST1 gene, whereas mutation of the PRPH2 gene is related to adult onset [3]. In older people, usually over 60, the occurrence of VLs is very common, defined as acquired VLs (aVLs), and related to several retinal pathologies: age-related macular degeneration (AMD), drusenoid accumulation, neovascularization, fibrosis, pachychoroid disease spectrum, vitreoretinal interface diseases with tractional disorders, toxic or paraneoplastic conditions, angioid streaks [2,4,5].

Despite different aetiologies, a common pathophysiologic pathway has been described. VLs are the result of the accumulation of waste material from the photoreceptor outer segments along with lipofuscin and melanolipofuscin, due to a process of retinal pigment epithelium (RPE) dysfunction, as shown by Arnold in her clinicopathologic study [6,7]. Recently, Sarraf and Feo have provided further details on the pathophysiology, emphasizing the possible role of the choroid in RPE dysfunction. In EDI-OCT studies, they reported the presence of aVL associated with both pachychoroid and leptochoroid. Specifically, the importance of the connection between increased or decreased choroidal thickness (CT) values and vitelliform lesions’ development and progression is increasingly recognized across these different conditions [8,9]. VLs are characterized by dynamic changes that may progress slowly over time. At first, the vitelliform material increases and then undergoes progressive resorption with the development of hypo-reflective spaces on OCT examination. Moreover, on SD-OCT scans, there may be changes in the outer retinal layers (external limiting membrane (ELM), ellipsoid zone (EZ)) up to atrophy of the RPE, associated with the onset of visual symptoms, such as reduced visual acuity [2]. The appearance of symptoms and changes in the characteristics of the VLs on retinal imaging are the basis of a sophisticated differential diagnosis workflow, necessary to distinguish a neovascular from a non-vascular subtype [10].

Multimodal imaging has become an essential tool for the diagnosis, monitoring and response to therapy of retinal disease [11]. Fluorescein angiography (FA) and indocyanine green angiography (ICGA) can be supportive in equivocal cases, although they are invasive examinations [12]. OCT angiography (OCTA) represents a key imaging modality for differentiating neovascular from non-neovascular hyperreflective spaces detected on structural OCT; nevertheless, its diagnostic performance may be limited in some cases, resulting in non-conclusive findings [13]. Moreover, retinal imaging analysis is limited by few factors: subjective analysis and the need for multiple examinations, which are not always readily available.

In recent years, radiomics has emerged as a quantitative, advanced high-performance medical image processing approach, leveraging various biomarker extraction and artificial intelligence (AI) techniques to create diagnostic and prognostic models [14,15]. These technologies have attracted great interest in radiology, with an exponential growth in scientific publications [16]. This has been matched by an increasing availability of AI-driven tools in clinical practice, with growing adoption by physicians [17]. The first publications on the application of radiomics in ocular imaging appeared in the 2020s. To date, the application is extensive, both in terms of the spectrum of pathologies and the availability of image types: OCT, OCTA, FAF, FA and ICGA for the posterior segment, OCT of the anterior segment, confocal microscopy for corneal endothelial cells [15].

Based on this trend, the objective of this study is to evaluate the role of radiomics in the analysis of OCT features of aVLs, to detect the presence of hypo-reflective retinal spaces and to distinguish between resorptive material in dynamic aVL changes and retinal fluid, using machine learning (ML) analysis.

## 2. Materials and Methods

### 2.1. Study Design

This retrospective, observational, monocentric study was conducted at the medical retina service of Eye Clinic of Federico II University, Naples, and included patients evaluated from March 2022 to January 2025. “The study was conducted in accordance with the Declaration of Helsinki. Ethical approval was obtained from the local Institutional Review Board of AOU Federico II of Naples (approval code: 06/19; approval date: 24 June 2019). The date of 24 June 2019 refers to the initial ethical approval. A subsequent favorable opinion was issued by the Campania 3 Ethics Committee on16 April 2025, under Protocol No. 27/2025 (minutes No. 6/25).” All included patients provided informed consent.

For the analysis, patients were stratified into two classes: class 1 corresponds to patients with a diagnosis of aVLs, and class 2, patients with a diagnosis of acute non-complicated central serous chorioretinopathy (CSCR). The diagnosis of CSCR served as the reference condition because it is characterized by the presence of subretinal fluid, which was essential for training the model to detect and differentiate retinal fluid from hypo-reflective space due to resorptive material.

Patients with aVLs were referred to our retinal service for further diagnostic evaluation from general ophthalmology clinics and were followed longitudinally. Given the retrospective design, OCT scans were not acquired at predefined intervals after the initial diagnosis. For each patient with aVLs, the OCT B-scan included in the radiomic analysis was selected based on the simultaneous presence of a hypo-reflective subretinal space and residual hyper-reflective vitelliform material, representing the clinically relevant resorptive stage investigated in this study. The selected scan could therefore correspond either to the first examination at our center or to a subsequent visit during longitudinal follow-up within the study period (2022–2025).

Exclusion criteria included: age < 18 years, axial length higher than 26 mm, the presence of clinically significant cataract, the presence of concomitant ocular or systemic diseases (such as diabetic retinopathy, uveitis, glaucoma), known other retinal disorders that could cause vitelliform lesions or retinal fluid (AMD, neovascularization, tractional pathologies, toxic or paraneoplastic syndromes), inherited dystrophies, previous retinal treatment. All patients underwent retinal multimodal imaging: multicolor imaging, FAF, SD-OCT and OCTA (Heidelberg Spectralis, Heidelberg Engineering) to perform the diagnosis and to rule out macular neovascularization. When OCTA findings were inconclusive due to imaging artifacts or media opacities, additional FA and/or ICGA (Heidelberg Spectralis, Heidelberg Engineering) were performed. Only uncomplicated aVL cases with no evidence of MNV were included in the study.

### 2.2. Scan Protocol

All SD-OCT examinations were acquired using Spectralis OCT (SPECTRALIS; Heidelberg Engineering, Inc., Heidelberg, Germany) with a 6 × 6 mm Dense Volume scanning protocol centered on the fovea, consisting of 49 B-scans. Poor-quality scans, defined by a signal strength index ≤15, were excluded [18].

### 2.3. Radiomics Features Extraction

For each patient, only one eye was included in the radiomic analysis. In unilateral cases, the affected eye was selected, whereas in bilateral cases, the right eye was systematically selected. From each included eye, a single B-scan displaying the target lesion was selected from the OCT volume for radiomic analysis. In the acute CSCR group, the selected B-scan contained subretinal fluid, whereas in the aVL group, it showed a hypo-reflective subretinal space associated with residual hyperreflective vitelliform material.

**Figure 1:**
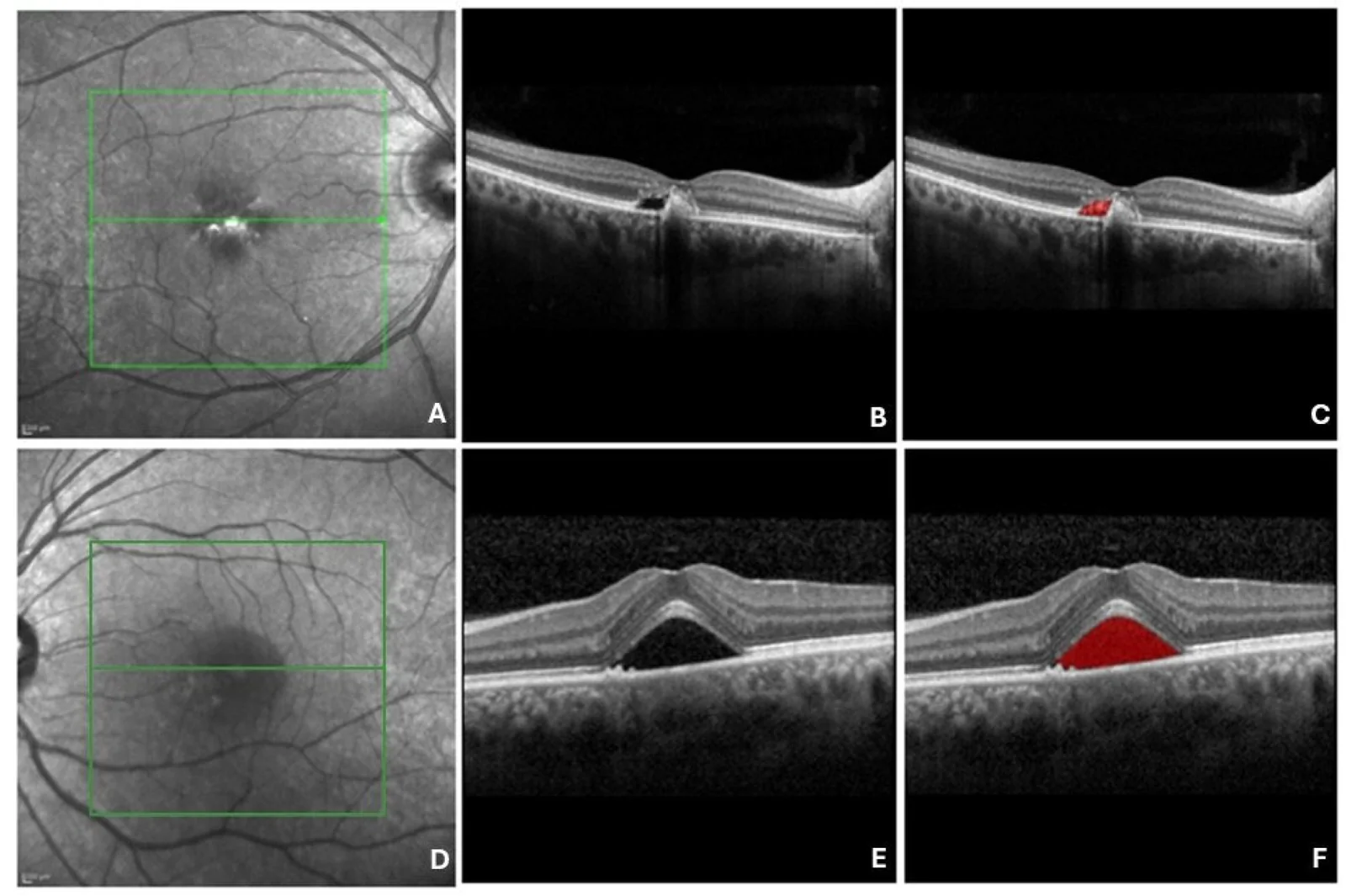
Example of manual segmentation of an acquired vitelliform lesion (**A**–**C**) and an acute central serous chorioretinopathy (**D**–**F**), on spectral domain optical coherence tomography. (**A**): near-infrared image; (**B**): corresponding B-scan SD-OCT; (**C**): region of interest (ROI—in red) corresponding to the hypo-reflective subretinal space; hyperreflective vitelliform material and surrounding normal retinal tissue were not included in the ROI, which was segmented with the ITK image segmentation software. (**D**): near-infrared image; (**E**): corresponding B-scan SD-OCT; (**F**): region of interest (ROI—in red) presenting the subretinal fluid due to CSCR, segmented with the ITK image segmentation software.

As a preliminary step, OCT images were exported as display images in PNG format, then underwent conversion from RGB to grayscale format, using the dedicated function in the PILLOW (v11.1) Python package, with default settings. Subsequently, three readers (M.C, E.G., M.S.) independently annotated each exam by manually delineating the regions of interest (ROI), corresponding to hypo-reflective spaces visible on structural OCT. Radiomic feature extraction was restricted to the manually segmented hypo-reflective ROI; residual hyperreflective vitelliform material and surrounding retinal tissue were not included in the analysis. The ITKSnap image segmentation software (v.3.8) was employed for this task (shown in Figure 1).

The multiple segmentations were necessary to allow feature stability analysis within the feature selection pipeline, while only the feature vector derived from the segmentation set generated by reader M.C. was used for the subsequent feature-selection steps and for model fitting and testing. Thus, each patient contributed only one feature vector to the final machine-learning dataset and the alternative reader segmentations were used exclusively for the assessment of feature robustness.

The resulting lesion masks were paired with corresponding grayscale OCT images to allow radiomic feature extraction with PyRadiomics (v3.0.1), which is an Image Biomarker Standardization Initiative-compliant software (https://pyradiomics.readthedocs.io/en/latest/index.html, accessed on 13 September 2026). This package performs both image preprocessing and biomarker extraction within the same process. In detail, resampling was applied with a 3 × 3 pixel kernel, followed by gray-level normalization (mean +/− 3 standard deviations), rescaling (factor = 100) and voxel array shift (+300). The result is an expected distribution of gray level values within the image in the 0–600 range. Then, gray level discretization (fixed bin width = 3) was applied. Together with the original images, preprocessed as described above, filtered images were also added to the extraction process to improve the signal-to-noise ratio of radiomic features. The filters consisted of a Laplacian of Gaussian edge enhancement (sigma values = 3, 4, 5) and wavelet decomposition (all high- and low-pass filter combinations along the *x* and *y* axes). Finally, morphologic (shape2D), first-order, and textural features were obtained from each lesion. The higher-order texture features are based on typical matrix calculations implemented in PyRadiomics. In summary, these include parameters derived from the region of interest using the gray-level co-occurrence matrix (GLCM), which describes distribution functions for gray-level values of pixel pairs; the level size zone matrix (GLSZM), quantifying pixel value zones (i.e., pixels sharing the same value); the level run length matrix (GLRLM), capturing gray-level run (i.e., consecutive pixels with identical value) characteristics and gray level dependence matrix (GLDM), focused on pixel value dependency from a center voxel within a pre-determined distance. Additional details on the feature classes and mathematical definitions for each feature are available through the online documentation (https://pyradiomics.readthedocs.io/en/latest/features.html, accessed on 13 September 2026).

### 2.4. Machine Learning Pipeline

All lesions were randomly split into training and test sets with a 75:25 ratio (using the model: sklearn.model_selection.train_test_split.html). Training data was used for feature selection, while test data was only transformed and processed at the time of inference, as described below, to avoid any risk of information leakage.

**Figure 2:**
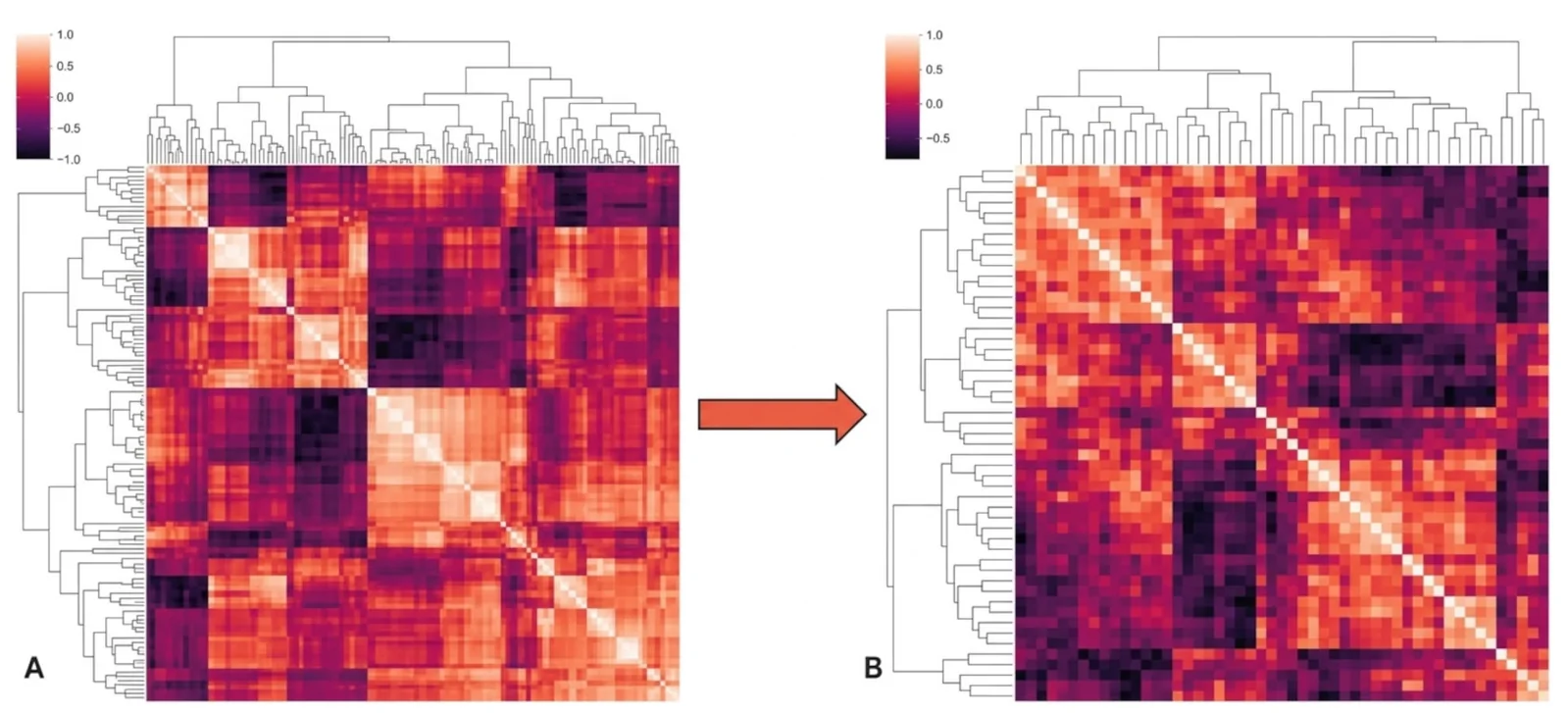
Clustermap of radiomics features correlation before (**A**) and after (**B**) selection with r > 80.

First, a two-way mixed-effects, absolute agreement, single-reader Intraclass Correlation Coefficient (ICC) model was employed to calculate feature robustness in relation to the manual segmentation process. Specifically, a 0.75 threshold for the ICC value was selected to identify reproducible features. Then, scaling was applied to eliminate a potential source of bias due to different value ranges across biomarkers. A MinMax (range = 0–1) scaler was therefore fitted on training data alone and used to transform both training and test data. Uninformative features were eliminated by applying a variance threshold (= 0.01) filter, while redundancy was addressed by calculating a pairwise, absolute value Pearson correlation matrix across all remaining features. A threshold of 0.8 was indicative of high inter-correlation, leading to the removal of one of the redundant features (shown in Figure 2).

At this point, class imbalance was addressed by using minority class oversampling with the Synthetic Minority Over-sampling Technique (k = 5). SMOTE was applied exclusively to the training set after dataset partitioning and was never applied to the held-out test set. Its purpose was to reduce the imbalance between the two diagnostic classes during model development.

The final step in feature selection employed the WEKA subset evaluator to balance feature informativeness and relative redundancy based on correlation to the class labels.

**Figure 3:**
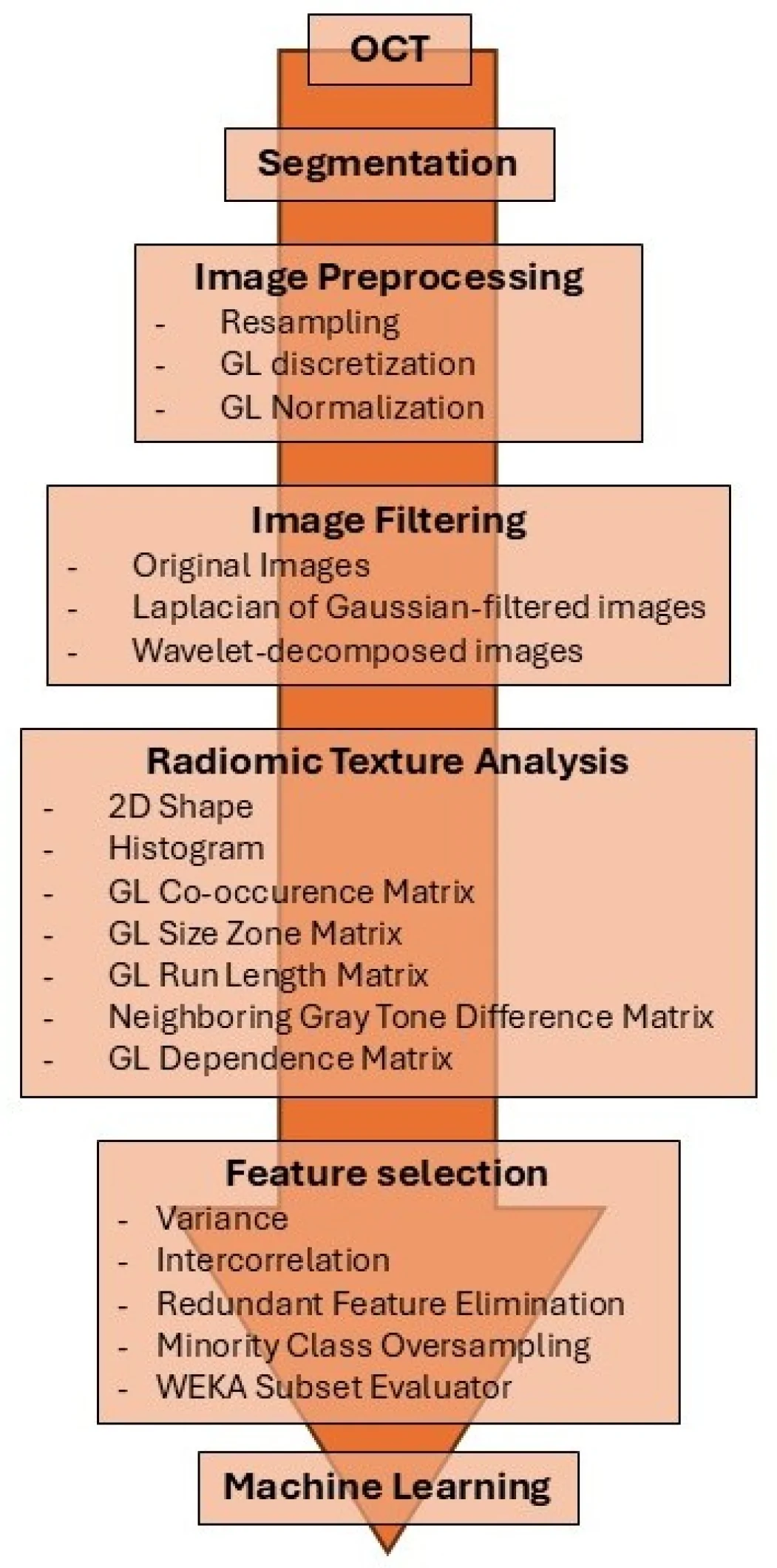
Radiomic workflow pipeline.

The resulting dataset was used to train a ridge-based LogisticRegression model, whose performance was assessed on the training data with 10-fold cross-validation and on the held-out test set. Common accuracy metrics were calculated based on the resulting confusion matrix. The analysis was conducted employing the “irr” (v0.84.1) R package, “scikit-learn” (v1.6) Python package and WEKA data mining platform (v3.9.6). The described radiomics workflow pipeline is illustrated in Figure 3.

## 3. Results

Based on inclusion and exclusion criteria, 99 patients were enrolled in the study. Demographic data are reported in Table 1.

**Figure 4:**
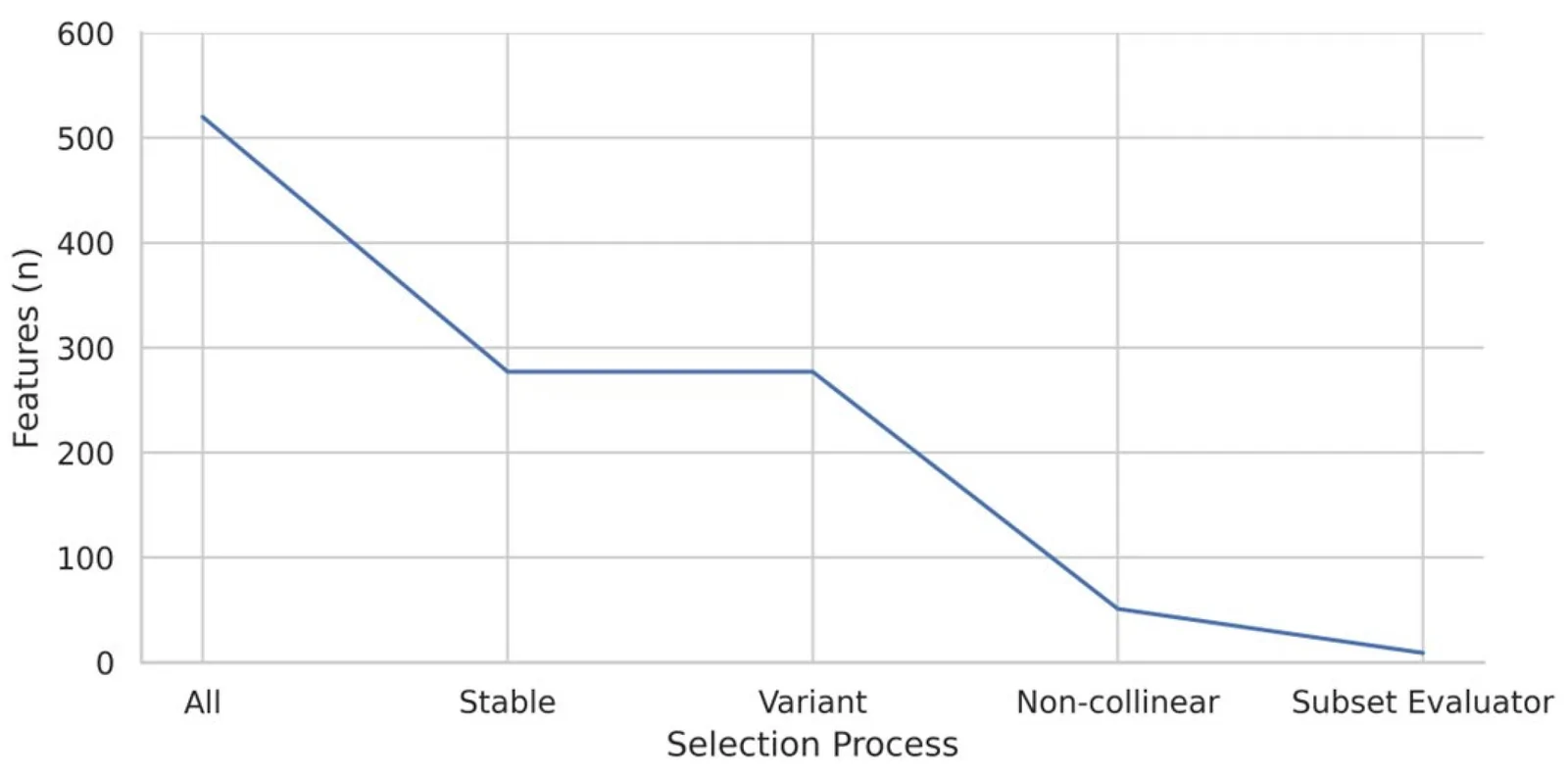
Plot depicting parameter number (*y*-axis) reduction during the various feature selection steps (*x*-axis).

After data partitioning, the 99 patient-associated B-scans were randomly partitioned into the training and held-out test sets at a 75:25 ratio, corresponding to 74 and 25 patients, respectively. No fellow-eye or repeated-scan observations from the same patient were included across different dataset partitions. From the B-scans, a total of 520 features were extracted. During the feature selection process, 277 resulted in stable features after ICC calculation, 277 were informative after variance analysis and 51 were not highly correlated with each other (shown in Figure 4).

**Figure 5:**
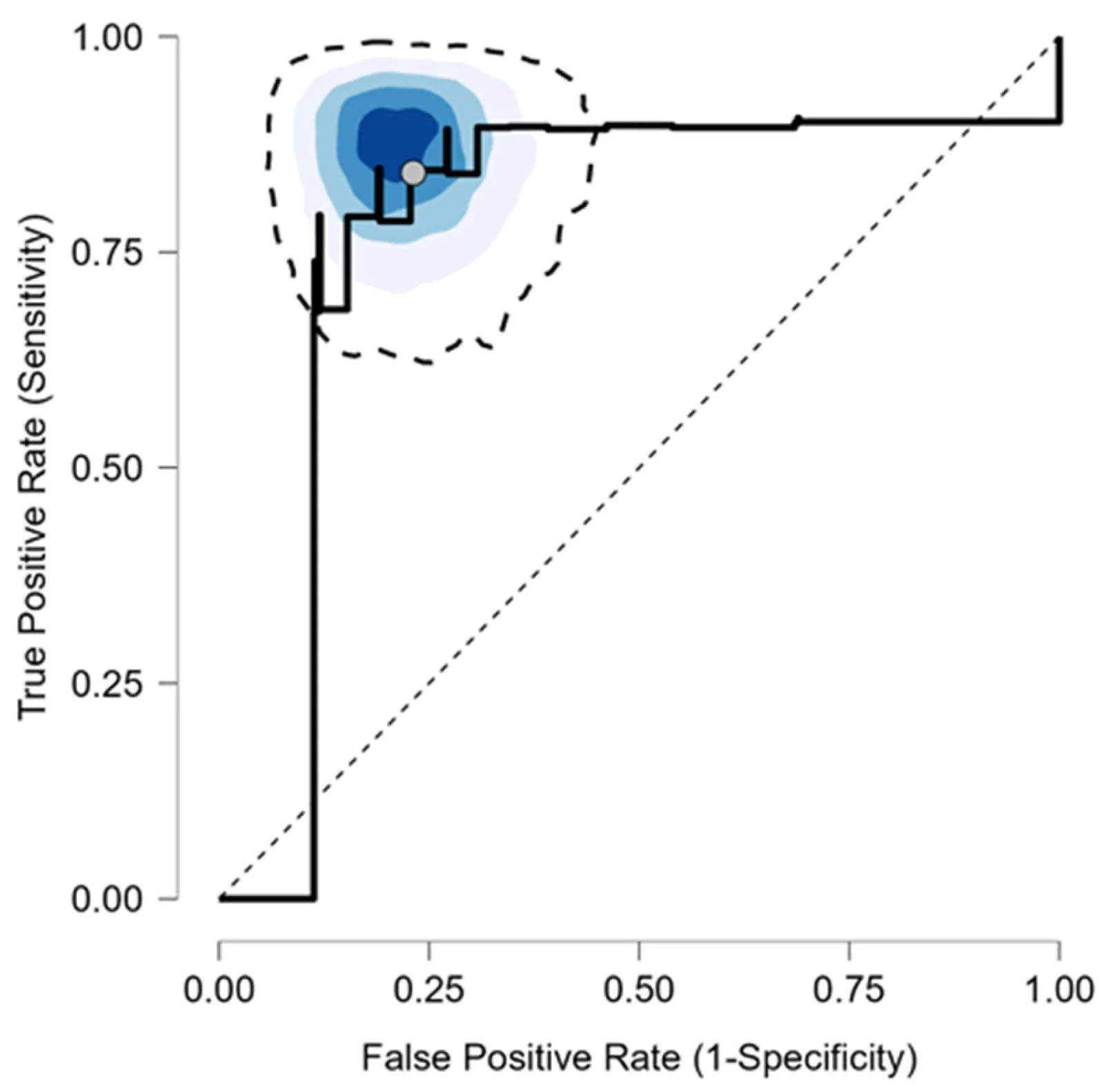
Receiver operating characteristic (ROC) curve of the radiomic classifier. The solid black line represents the ROC curve, while the diagonal dashed line represents the reference line for random classification. The highlighted point indicates the selected operating point of the classifier. The model achieved an AUC of 0.92 in the held-out test set. The solid black line represents the ROC curve across decision thresholds. The grey circle indicates the summary operating point. The blue shaded region depicts the bivariate posterior density distribution centered around the summary point, with darker regions indicating higher probability density. The dashed outer contour bounds the 95% confidence region. The diagonal dashed line denotes chance-level performance.

**Figure 6:**
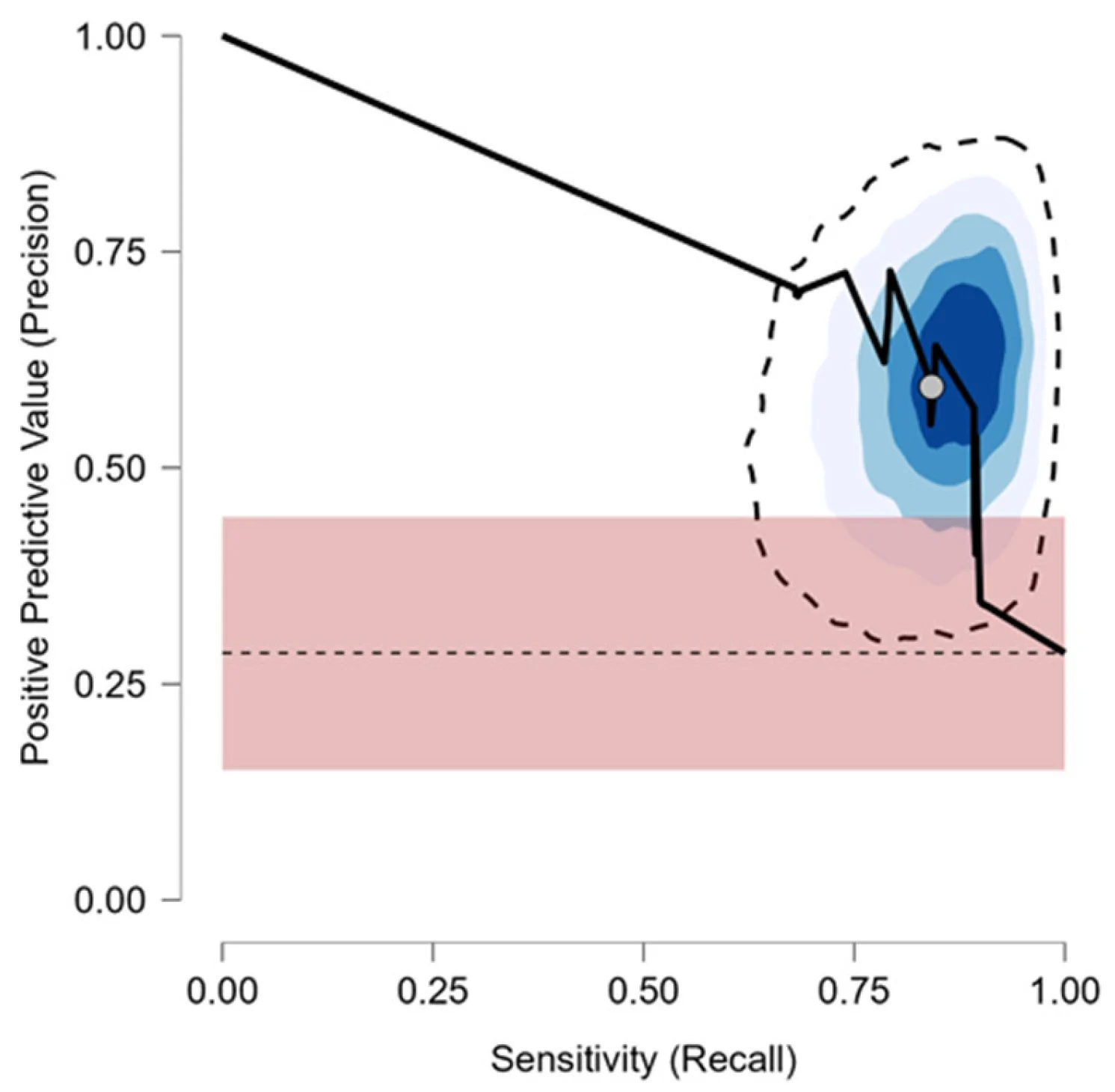
Precision–Recall (PR) curve of the radiomic classifier in the held-out test set. The solid black line represents the relationship between precision (positive predictive value) and recall (sensitivity) across different classification thresholds. The model achieved an area under the precision–recall curve (PR AUC) of 0.92 in the held-out test set. The solid black line represents the PRC showing the trade-off between Positive Predictive Value (Precision) and Sensitivity (Recall) across decision thresholds. The grey circle denotes the summary operating point. The blue shaded region depicts the bivariate density distribution centered at the summary point, with darker shades indicating higher probability density. The outer dashed contour bounds the 95% confidence region. The horizontal dashed line marks the baseline pre-test probability, representing chance-level performance, with its uncertainty bounded by the pink shaded region.

The SubsetEvaluator identified a final set of 9 features to be employed for model training (original_shape2D_SphericalDisproportion, original_firstorder_Range, original_glcm_DifferenceEntropy, log-sigma-3-0-mm-3D_firstorder_Skewness, log-sigma-5-0-mm-3D_firstorder_Skewness, log-sigma-5-0-mm-3D_glcm_ClusterTendency, log-sigma-5-0-mm-3D_glcm_Idn, log-sigma-5-0-mm-3D_glrlm_RunVariance, wavelet-H_firstorder_Kurtosis). Based on these, the LogisticRegression model was able to obtain an 86.5% accuracy on the training data, with a positive predictive value (PPV) and negative predictive value (NPV) of 87%, an area under the ROC curve (AUC) of 0.89 and an area under the Precision–Recall curve (PRC) of 0.86 (Figure 5 and Figure 6).

In the test set, the model was able to achieve an 80% accuracy, 92% NPV and 67% PPV. The AUC value was 0.92, while the PRC area was 0.92 (Table 2). The confusion matrix of the test dataset is reported in Table 3.

## 4. Discussion

To the best of our knowledge, this is the first study to assess the performance of radiomics analysis to classify acquired vitelliform lesions (aVLs) with collapsed subretinal material by distinguishing hypo-reflective subretinal spaces between reabsorbed material and true subretinal fluid, using an AI-based approach. Although aVLs have been extensively studied using SD-OCT and the progressive evolution from “egg yolk” stage to atrophy has been well characterized, the differential diagnosis between resorbed material and the presence of perilesional fluid secondary to neovascular complications remains challenging [19]. In a large series by Balaratnasingam and colleagues, patients diagnosed with aVLs had poor visual outcome due to macular neovascularization in 12% of cases and atrophy in 44% [12]. In both cases, SD-OCT examination typically revealed a hyporeflective area near the hyperreflective subretinal material, often associated with vision loss. In such cases, SD-OCT analysis alone may not be decisive, defining the complementary role of OCTA, which enables direct visualization of the vascular network. To overcome the limitations associated with additional retinal imaging examinations, such as OCTA, or invasive examinations, such as FA and ICGA, radiomics analysis was applied to an SD-OCT dataset of patients diagnosed with non-complicated aVLs and compared with a group of patients diagnosed with acute CSCR. The control group was selected to enable the model to be trained on the detection of retinal fluid, as acute CSCR is characteristically associated with the presence of subretinal fluid. The dataset was therefore used to train the algorithm to differentiate the two different conditions. This distinction provides an indirect indication of possible neovascular complications in aVLs.

Radiomics is an emerging field of medical image analysis that aims to extract many features from standard clinical images using advanced computer algorithms. The hypothesis underlying radiomics is that these features, such as shape, texture, intensity and spatial relationship, could provide information that is not appreciable to the naked human eye [15]. In recent years, radiomics has been extensively applied to the field of oncology, where its potential to enhance clinical decision-making, patient management, and clinical outcomes has been well demonstrated [20]. Beyond oncology, radiomics entered the field of ophthalmology in 2021, enabling the extraction of a wide range of quantitative features from routinely acquired images. To date, radiomics analysis has been increasingly employed across a broad spectrum of retinal disorders, including AMD and diabetic retinopathy, due to their high prevalence and social burden. More recently, its use has also extended to vitreoretinal and retinal vascular diseases [21,22,23]. Although radiomics in ophthalmology is still in its early stages, its integration with AI and ML frameworks appears to advance precision diagnostics, risk stratification, and individualized treatment strategies, contributing to the development of more personalized ophthalmic care.

In this context, our study was designed to explore these capabilities. In the training set, the classifier achieved an accuracy of 86.5%, with balanced positive and negative predictive values (87% each), indicating good capability to correctly identify both positive (i.e., retinal fluid) and negative (reabsorbed material) lesions. The high AUC (0.89) and precision-recall score (0.86) further support the model’s global discriminative ability and its robustness in potentially imbalanced datasets. On the internal held-out test set, model performance slightly decreased but remained clinically meaningful, with an accuracy of 80%. The observed reduction in performance is consistent with the expected variability of unseen data and suggests that the model retains reasonable performance in the held-out test set. The validity of the test is also supported by the excellent AUC (0.92) and PRC (0.92) values maintained in the validation set. It is worth stressing the importance of the negative predictive value, which is particularly high (92%) in the test set, highlighting the model’s strong ability to exclude false negatives, a desirable feature for screening applications. The reduction in positive predictive value from 87% in the training set to 67% in the held-out test set indicates a higher rate of false-positive fluid classifications and warrants cautious interpretation of positive model outputs. A positive classification should not be regarded as diagnostic of true subretinal fluid or underlying MNV, but rather as an indication for further assessment with multimodal imaging, particularly OCTA. Overall, these findings indicate that the model distinguishes well between the two different OCT patterns, with strong potential as a supportive complementary decision-support tool, rather than as a standalone diagnostic method. From a clinical perspective, the proposed model should be regarded as a complementary decision-support tool, rather than an alternative to OCTA or dye-based angiography. A model output suggestive of fluid could increase suspicion of an exudative complication and prompt targeted multimodal assessment, particularly OCTA and, where clinically indicated, FA or ICGA. At this stage of the development, model output should not, however, be considered sufficient to establish a diagnosis of MNV. Classification as resorptive material could provide additional support for a non-exudative interpretation, particularly in view of the high NPV observed in the test set.

Although our findings are promising, the study has several limitations. These include the retrospective design, the small sample size, the manual segmentation of the ROI, and the challenges related to both statistical analysis and interpretation of results, which require specific clinician expertise. To address the issue of manual segmentation and to reduce the variability, each OCT examination was independently segmented by three readers, and feature reproducibility across segmentations was assessed using a two-way mixed-effects, absolute-agreement, single-reader ICC model. Only radiomic features with an ICC > 0.75 were retained for subsequent analysis, resulting in 277 of the initial 520 features meeting the predefined reproducibility criterion. Nevertheless, automated or semi-automated segmentation approaches should be investigated in future studies to further improve reproducibility and facilitate translation of the proposed workflow into clinical practice.

Despite these limitations, the strength of our study lies in the robust design of the radiomics model and the rigorous statistical analysis. All recommended steps were followed to ensure the development of a robust and compliant radiomics model, in line with the best practice guidelines defined by the European Society of Medical Imaging Informatics [24].

Future larger prospective, multicentre studies with independent external validation cohorts will be required to validate the diagnostic performance of the model and to assess its impact on clinical decision-making, particularly its ability to reduce diagnostic uncertainty and unnecessary additional imaging without increasing the risk of missed neovascular complications.

In conclusion, these preliminary findings underscore the promising role of AI-driven radiomics applied to OCT imaging in characterizing intrinsic features of acquired vitelliform lesions. By extracting high-dimensional quantitative biomarkers, this approach may enhance the diagnostic precision of retinal imaging, improving the ability to differentiate reabsorbed material from true retinal fluid, and analyzing a single exam on structural OCT. It should be emphasized that the proposed model is still at an early stage of development and is not intended to replace multimodal retinal imaging. Rather, it is designed to complement the interpretation of SD-OCT by providing additional diagnostic support in clinical settings where multimodal imaging is not readily available or access to advanced imaging resources is limited.

## Figures and Tables

**Table 1:** Demographic data of population study.

	aVLs	CSCR
Patients (N.)	60	34
Age (Mean ± SD)	45 ± 5	40 ± 5
F:M (%)	60%	65%

**Table 2:** Detailed accuracy metrics of the datasets. PPV: positive predictive value; NPV: negative predictive value; ROC: Receiver Operating Characteristic; PRC: Precision–Recall curve.

	Accuracy (%)	PPV (%)	NPV (%)	ROC Area	PRC Area
Training set	86.7	87	87	0.89	0.86
Test set	80	67	92	0.92	0.92

**Table 3:** Confusion matrix for the test group.

		Predicted Label	
		Resorptive	Fluid
Actual label	Resorptive	12	4
	Fluid	1	8

## Data Availability

The original contributions presented in this study are included in the article. Further inquiries can be directed to the corresponding author.
